# Different Proteins as Biomarkers for Sac Shrinkage After Endovascular Aortic Repair of Abdominal Aortic Aneurysms

**DOI:** 10.3390/jcdd11110374

**Published:** 2024-11-20

**Authors:** Alexander Zimmermann, Daniela Reitnauer, Yankey Yundung, Anna-Leonie Menges, Lorenz Meuli, Jaroslav Pelisek, Benedikt Reutersberg

**Affiliations:** Department of Vascular Surgery, University Hospital Zurich, 8091 Zurich, Switzerland; daniela.reitnauer@usz.ch (D.R.); anna-leonie.menges@usz.ch (A.-L.M.); lorenz.meuli@usz.ch (L.M.); jaroslav.pelisek@usz.ch (J.P.); benedikt.reutersberg@usz.ch (B.R.)

**Keywords:** aneurysm diameter, endovascular aneurysm repair, proteomics, neurogranin, casein alpha S1, calpastatin, SCUBE3, ubiquitin-conjugating enzyme E2

## Abstract

Background: This study aims to identify circulating biomarkers by using proteomic analysis associated with sac shrinkage or expansion in patients undergoing endovascular aneurysm repair (EVAR) for abdominal aortic aneurysms (AAAs). Methods: Plasma samples were analysed from 32 patients treated with EVAR between 10/2009 and 10/2020. Patients were divided into two groups based on postoperative sac behaviour: sac shrinkage (≥5 mm reduction) and no shrinkage (stabilisation or expansion). Proteomic analysis was performed using high-resolution liquid chromatography-tandem mass spectrometry (LC-MS/MS), with abundant protein depletion to enhance the detection of low-abundant proteins. Results: Of the 32 patients, 20 exhibited sac shrinkage, and 12 showed no shrinkage. Proteomic analysis identified 632 proteins, with significant differential abundance observed after adjusting for relevant clinical parameters. Notably, neurogranin (NRGN) levels were significantly associated with hypertension and smoking, while casein alpha S1 (CSN1S1) levels varied with statin use. Differentially abundant proteins related to aortic diameter included calpastatin, SCUBE3, and ubiquitin-conjugating enzyme E2, among others. Conclusions: Proteomic profiling revealed distinct biomarker patterns associated with sac behaviour in EVAR-treated AAA patients. These findings suggest potential therapeutic targets for enhancing EVAR outcomes and underscore the need for further investigation into the biological mechanisms underlying aneurysm sac shrinkage and stability.

## 1. Introduction

An abdominal aortic aneurysm (AAA) is a potentially life-threatening condition characterised by the localised dilation of the abdominal aorta. In the meantime, endovascular aneurysm repair (EVAR) has become the most commonly performed invasive method for treating abdominal aortic aneurysms in Western countries, ahead of open surgery [1]. Nevertheless, the long-term outcomes of EVAR remain a matter of concern, particularly in instances where the aneurysm sac remains large or continues to expand following the procedure [2].

Sac shrinkage following EVAR in patients with AAAs is considered a key indicator of successful treatment and reduced risk of complications [3,4]. A number of morphological factors, including the size and shape of the aneurysm neck and the presence of thrombus, as well as patient-specific factors such as statin therapy and biological factors such as inflammatory markers (neutrophil-to-lymphocyte ratio, NLR), have already been identified as significantly associated with the rate of sac shrinkage or growth [5,6]. In addition, studies have attempted to identify biomarkers of aneurysm sac progression before and after EVAR by using genetic profiling or by analysing limited sets of pre-selected proteins [7,8,9].

It is notable that there is a paucity of knowledge regarding the biological mechanisms that underpin sac shrinkage, particularly in the context of EVAR-treated patients without endoleaks. Endoleaks following EVAR can result in an increase in aortic diameter and, therefore, do not reflect the biological processes underlying either aortic sac shrinkage or stability/expansion in successfully implanted endografts [10].

Proteomics, the large-scale study of proteins, has emerged as a powerful tool for the identification of novel biomarkers and the elucidation of disease processes. The identification of these dysregulated proteins offers intriguing possibilities for future diagnostics and therapeutic interventions. Identifying specific proteins or pathways involved in sac shrinkage may provide new avenues for developing adjunctive treatments to enhance the effectiveness of EVAR and improve long-term outcomes for AAA patients.

The aim of this study is to identify potential circulating biomarkers that may be associated with aneurysm sac stability/expansion or shrinkage in patients who have undergone EVAR treatment.

## 2. Materials and Methods

### 2.1. Study Cohort

All patients who had undergone endovascular aortic repair (EVAR) for an infrarenal aortic aneurysm between October 2009 and October 2020 at the University Hospital of Zurich, Switzerland, were screened retrospectively for eligibility for this study (*n* = 57). The inclusion criteria were age >50 years at the time of EVAR and availability of a signed informed general consent for research as by the Swiss Federal Act on Research involving Human Beings [11]. Individuals who met any of the following criteria were excluded from the study (*n* = 25): participation in other clinical studies with the potential to interfere with the project outcome; rejected informed consent; hereditary diseases (e.g., Loeys–Dietz syndrome, Marfan syndrome, Ehlers–Danlos syndrome); inflammatory diseases; any kind of endoleak in the follow-up period; or a follow-up period of less than two years.

The local Ethics Committee (Cantonal Ethics Committee Zurich, Switzerland; BASEC-Nr. 2020-00378) approved the sample collection and analysis procedure as part of the Swiss Vascular Biobank in 2020. This study and the specific data analysis were approved by the local Ethics Committee (BASEC-No 2022-02033) in 2022 and adhered to the tenets of the Declaration of Helsinki.

The patients were identified using the institutional clinical information system (KISIM 5.1.0.3; CISTEC AG, Zurich, Switzerland). Baseline clinical data were collected, including sex, age, comorbidities, details of medication, and operative details (diameter and stent graft used).

### 2.2. Follow-Up Regimen and CT Measurements

Patients were typically advised to be followed up at three, six, and twelve months, with annual follow-ups thereafter. In the event of an endoleak, stent graft migration, or sac enlargement being identified, a CTA was conducted at six-month intervals to ascertain the necessity for reintervention. Two reviewers (AZ and DR) analysed the CTA data using a 3-dimensional workstation (XERO Viewer 8.1.2, Agfa HealthCare N.V., Mortsel, Belgium). The diameter of the abdominal aorta was measured on the cross-sectional plane perpendicular to the centreline based on the adventitia. The preoperative CTA was employed as the baseline CTA and compared with the 2-year follow-up CTA. The changes in the maximum diameter were used to classify aneurysm sac behaviour.

### 2.3. Definition of Aneurysm Sac Behaviour

Following the guidelines established by the Society of Vascular Surgery [12], sac shrinkage was defined as a reduction of ≥5 mm at the 2-year follow-up compared to the preoperative maximum AAA diameter. For analysis, the cohort was divided into two groups: those with sac shrinkage of ≥5 mm (group “Sh”), and those without (group “I”), which included all other patients with either sac stabilisation or sac expansion.

### 2.4. Blood Sampling and Protein Isolation

Plasma samples were collected between October 2022 and May 2023 from patients identified in our clinical database who met all the inclusion and exclusion criteria. The blood sample was taken during a regular follow-up appointment and was obtained by puncturing a brachial vein. The patient was not instructed to fast before the appointment.

Further processing was carried out by immediate centrifugation (within 1 h) after the samples were collected in EDTA tubes (10 mL). Subsequently, plasma aliquots (1 mL each) were stored at −80 °C until further use. The proteins were isolated using a High-Select Abundant Protein Depletion Resin Kit (ThermoFisher Scientific, Zug, Switzerland) in order to facilitate the identification and quantitation of low-abundant proteins. The assay eliminates the most abundant proteins in plasma samples, including albumin, immunoglobulins, fibrinogen, haptoglobin, and transferrin, thus enhancing the sensitivity of proteome analysis.

### 2.5. Proteome Analysis of Plasma Samples

A proteomic analysis was conducted utilising high-resolution liquid chromatography-tandem mass spectrometry (LC-MS/MS). For protein identification and quantification, the FragePipe TMT proteomics pipeline was employed [13]. The protein matrix was subjected to filtration using a minimum of two peptides as the threshold. In order to perform the differential abundance analysis of the plasma proteins in the study groups, a number of steps were taken. Firstly, a linear model was applied to each protein using the R function lm to ascertain whether the observed protein abundances were consistent with the fitted peptide counts. Subsequently, the difference in the protein abundancies between the study groups was calculated. The null hypothesis was that the proteins were not significantly different. The estimated detection limit was calculated as the mean of the 1% smallest group averages. Subsequently, variance shrinkage was performed to enhance the statistical power of the analysis per the recommendations set forth by Smyth [14]. Finally, the false discovery rate (FDR) was computed using the Benjamini–Hochberg procedure [15], with an FDR threshold of 0.1, to determine whether there were any statistically significant differences in the individual proteins between the study groups.

### 2.6. Statistical Analysis

Categorical variables were presented as numbers (percentages), while continuous variables were expressed as the median and range. The categorical/nominal variables of the demographic data and early outcomes were compared using Fisher’s exact test; the metric variables were analysed using the non-parametric Mann–Whitney U-test. All *p*-values were deemed statistically significant at a 5% alpha level. Correlation analysis to obtain non-parametric Spearman correlation coefficients was conducted using SPSS 23.0 (IBM Corp. Released 2015. IBM SPSS Statistics for Windows, Version 23.0. Armonk, NY, USA: IBM Corp.). A multivariable logistic regression analysis was conducted to adjust the protein abundance for the selected clinical parameters. Due to the requirement of logistic regression to use only dichotomous variables (yes/no, 1/0), the nominal variables were dichotomised using a cut-off value, resulting in two groups with a similar number of study samples. The age of the subjects was divided into two groups, with a cut-off of 70 years. Creatinine levels were also divided into two groups, with a cut-off of 90 mmol/L. Finally, the diameter of the subjects was divided into two groups, with a cut-off of 50 mm.

Volcano and violin plots were used for visualisation: volcano plots are a type of scatterplot to visualise statistical significance (*p*-value) versus fold change in large datasets, while violin plots show the distribution, density, and summary statistics of a dataset across different categories.

## 3. Results

### 3.1. Patient Clinical Characteristics

After searching the clinical database and applying the inclusion and exclusion criteria, a total of 32 patients were included in this study. Of these, 20 (62.5%) patients showed sac shrinkage 2 years after EVAR implantation (group “Sh”), and 12 (37.5%) patients showed a stable or increasing aneurysm sac (group “I”). There were no aortic or stent graft-related events during follow-up. The median age was 73 years (range 56–89 years), with no significant difference between the two groups. Only male patients (100%) could be recruited for this study. Furthermore, the prevalence of comorbidities and the type of medication being taken were found to be comparable between the two groups. The median diameter of the aneurysms prior to the procedure was 59 mm without significant differences between the study groups. In contrast, the diameter at follow-up CTA was found to be significantly reduced in the shrinkage group (median 47 mm) compared to patients with stable or expanded aortic diameter (median 57) (*p* = 0.026). Table 1 presents a comprehensive overview of all patient characteristics.

### 3.2. Proteome Analysis of Plasma Samples

Abundant protein depletion enabled the identification of low-abundant proteins in the plasma of the study patients. The overall number of detected proteins was 632. The average number of identified proteins per sample was 567 (range 530 to 620), with high protein abundance in all samples.

**Figure 1:**
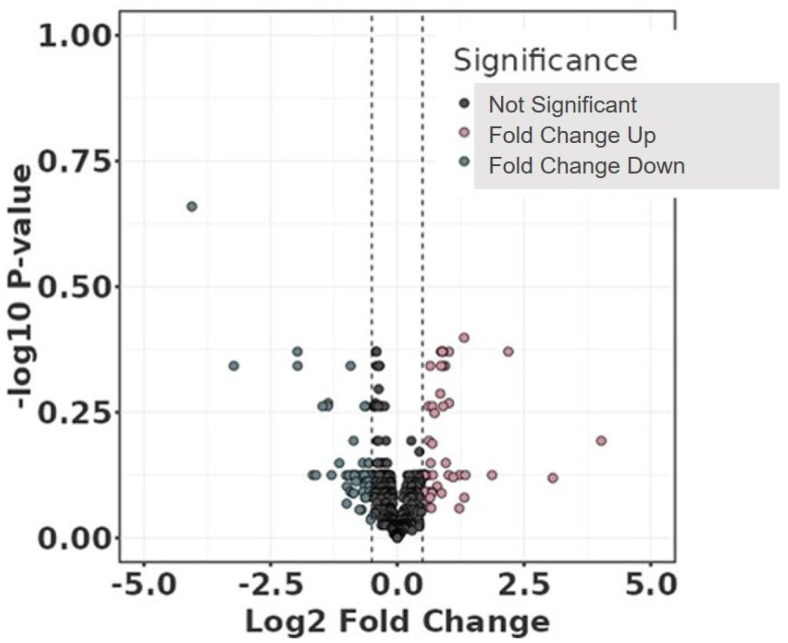
Volcano plot comparing study groups with (Sh) and without (I) shrinkage without any adjustment. The plot shows −log10 transformed the false discovery rate as a function of the difference between groups. The broken lines show 0.5 log2 fold change.

**Figure 2:**
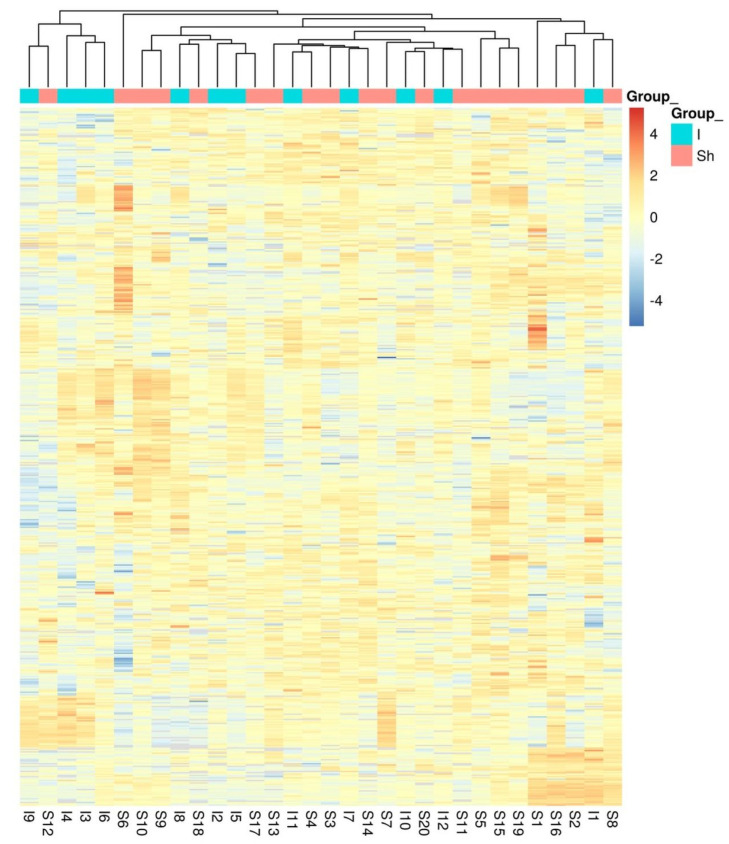
Protein abundance heatmap (rows indicate proteins; columns indicate samples) showing the row scaled log2 transformed protein abundance value. Co−clustering (hierarchical complete linkage, Euclidean distance) of samples and proteins was used. Study groups with (Sh) and without (I) shrinkage.

The differential abundant protein analysis between the study groups is displayed in the volcano plot (Figure 1), and the clustering of the individual samples is shown in the protein abundance heatmap (Figure 2). Regarding the comparison between the study groups and FDR < 0.1 without any adjustment, no significant differences were observed for any plasma protein. The clustering of the individual plasma samples (Figure 2) demonstrates the high heterogeneity of the individual samples. The study group with aortic sac shrinkage showed more accumulation on the right side of the heatmap than the non-shrinkage patients on the left side. However, individual samples were found on both sides of the heatmap. 

**Figure 3:**
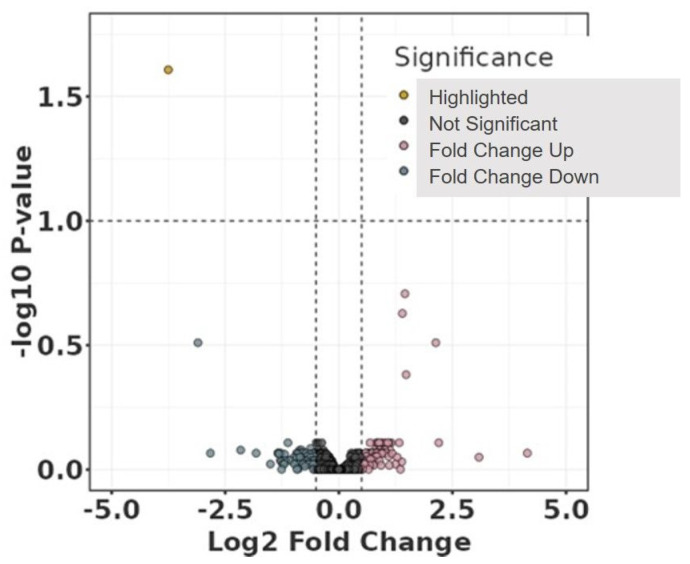
Volcano plot comparing study groups with (Sh) and without (I) shrinkage adjusted for clinical parameter hypertension, cardiovascular disease, or smoking. The plot shows the −log10 transformed false discovery rate as a function of the difference between the groups. The broken lines show the 0.5 long2 fold change.

**Figure 4:**
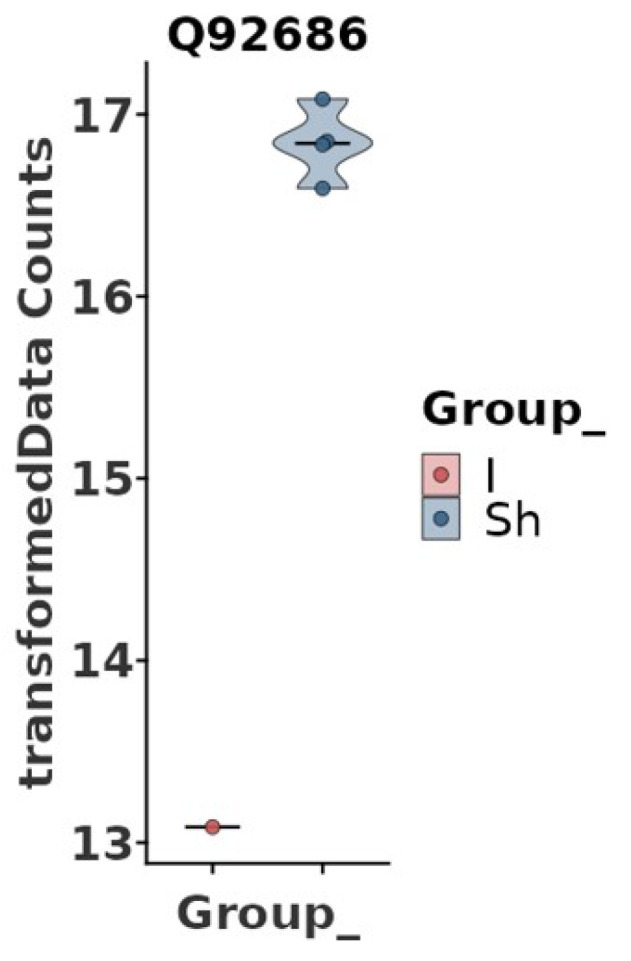
Violin box plot comparing study groups with (Sh) and without (I) shrinkage adjusted for clinical parameter hypertension, cardiovascular disease, or smoking. The abundance of neurogranin (NRGN) was significantly higher in the shrinkage group compared to the idem group (false discovery rate < 0.1).

**Figure 5:**
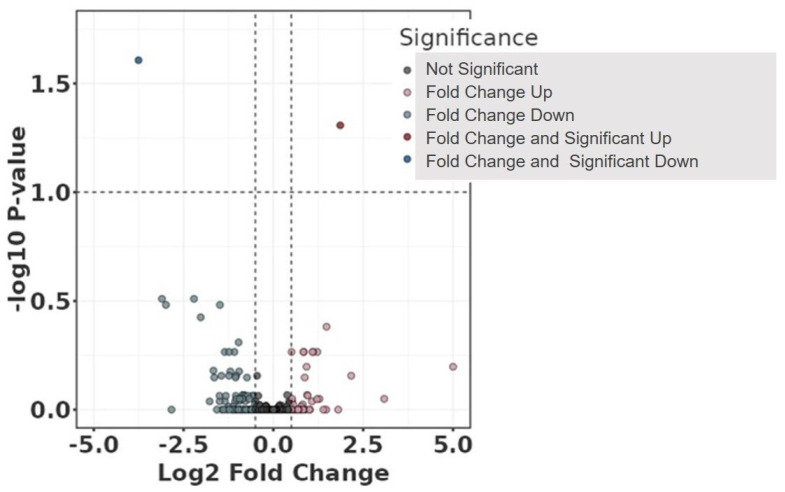
Volcano plot comparing study group with (Sh) and without (I) shrinkage adjusted for the clinical parameter statin. The plot shows the −log10 transformed false discovery rate as a function of the difference between groups. The broken lines show the 0.5 long2 fold change.

**Figure 6:**
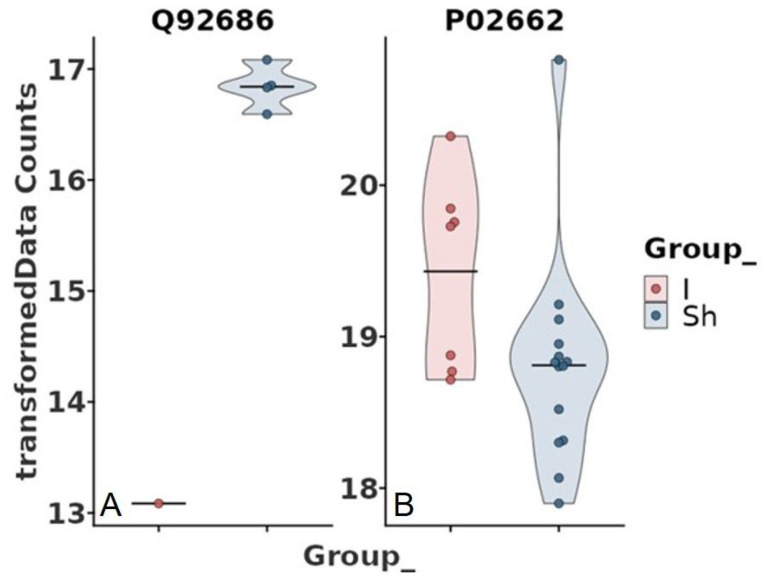
Violin box plots comparing study groups with (Sh) and without (I) shrinkage adjusted for the clinical parameter statin. (**A**) neurogranin (NRGN); (**B**) casein alpha S1 (CSN1S1), false discovery rate < 0.1.

**Figure 7:**
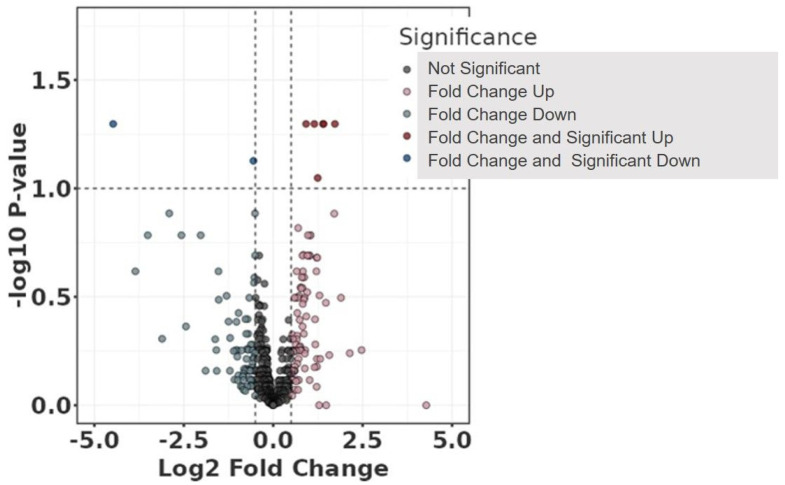
Volcano plot comparing study group with (Sh) and without (I) shrinkage adjusted for the clinical parameter diameter. The plot shows the −log10 transformed false discovery rate as a function of the difference between the groups. The broken lines show the 0.5 log2 fold change.

**Figure 8:**
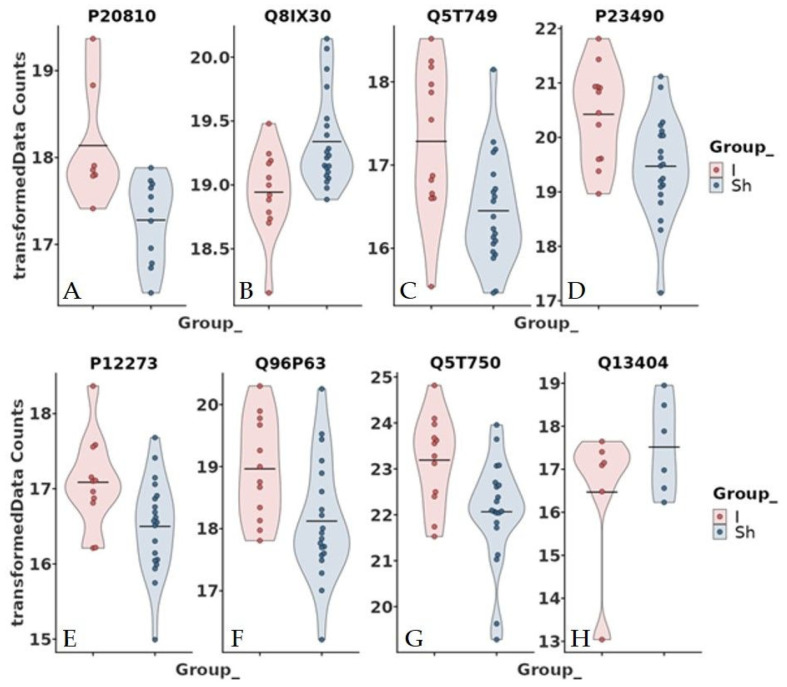
Violin box plots comparing study groups with (Sh) and without (I) shrinkage adjusted for the clinical parameter diameter. (**A**) calpastatin, (**B**) SCUBE3, (**C**) keratinocyte proline-rich protein (KPRP), (**D**) loricrin, (**E**) prolactin-inducible protein (PIP), (**F**) SERPINB12, (**G**) skin-specific protein 32 (C1orf68), and (**H**) ubiquitin-conjugating enzyme E2 (UBE2); false discovery rate < 0.1.

However, interesting results were found after adjustment for some of the clinical data (Table 1). Age, chronic obstructive pulmonary disease (COPD), diabetes mellitus (DM), chronic kidney disease (CKD), hyperlipidemia, antiplatelet therapy, anticoagulation, and beta-blocker were not associated with protein abundance among the study groups. In contrast, hypertension, cardiovascular disease (CVD), and smoking were significantly associated with the abundance of neurogranin (NRGN) (Figure 3 and Figure 4). Furthermore, adjustment for statin intake leads to differences in the concentration of NRGN and human casein alpha S1 (CSN1S1) (Figure 5 and Figure 6). Finally, the adjustment for diameter at the baseline (cut-off 50 mm) led to the discrimination of the following proteins within the plasma samples: calpastatin, SCUBE3, keratinocyte proline-rich protein, loricrin, prolactin-inducible protein, serpin B12, skin-specific protein 32, and ubiquitin-conjugating enzyme E2 (Figure 7 and Figure 8). All the results of the significantly abundant proteins following adjustment, including FDR and fold change, are summarised in Table 2.

**Figure 9:**
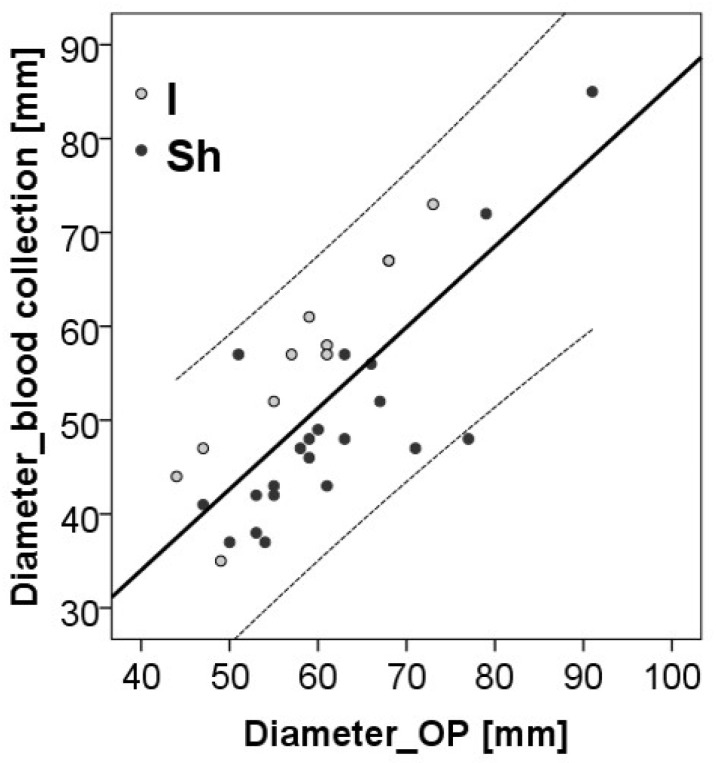
Correlation analysis of aortic sac diameter at the time point of the endovascular intervention and the time point of blood collection. The Spearman correlation coefficient was r = 0.701, *p* < 0.001. Study groups with (Sh) and without (I) shrinkage. OP: time point of EVAR.

In addition, due to the higher number of abundant proteins after the adjustment for aortic sac diameter at the baseline and the different time points in aortic sac measurement and blood sampling, we performed a correlation analysis between the diameter of the aorta at the time of EVAR intervention and the time of blood sampling (43 ± 27 months). A high correlation coefficient was observed with r = 0.754 and *p* < 0.001. A clear distinction between the study groups was found (Figure 9). Whereas the diameter of the shrinkage patients lay under the dividing line, most of the patients without shrinkage were found above the separating line.

## 4. Discussion

General considerations

The identification of plasma proteins positively or negatively associated with aortic sac shrinkage after EVAR may provide potential diagnostic and therapeutic targets that could enable improved outcomes.

Numerous clinical and morphological factors that can or might influence sac behaviour after EVAR have been identified to date [5,6]. This study showed no statistically significant differences for any of these previously identified baseline characteristics between patients with and without sac shrinkage. However, this must be seen in the light of the small size that hinders any firm conclusions.

Patients with endoleaks were excluded from this study because endoleaks with residual blood flow in the aneurysm sac significantly impact the aneurysm sac behaviour and may influence the long-term prognosis of EVAR [16,17]. This study provided data on proteins detected by proteomics in the blood that differed significantly between a post-EVAR group with sac shrinkage and a group without sac shrinkage, thereby excluding patients with endoleaks.

Neurogranin (NRGN)

Following adjustment for hypertension, CVD, or smoking, neurogranin was significantly different between the study groups. Neurogranin is a small calmodulin-binding protein abundantly expressed in several brain regions, and it also participates in the protein kinase C signalling pathway. NRGN is involved in synaptic plasticity, and increased levels have been observed in the cerebrospinal fluid of Alzheimer’s disease patients [18]. Interestingly, a recent study discovered that NRGN can also regulate the phenotypic switching of human aortic small muscle cells (SMCs) [19]. Furthermore, Laudanski et al. observed that patients after cardiac surgery exhibited persistent peripheral and neuronal inflammation, blood vessel remodelling, and the depletion of neuroprotective factors three months post-procedure [20]. Persistent or long-lasting inflammation may also develop after EVAR treatment, particularly in patients with hypertension, CVD or smoking, leading to a significant reduction in NRGN in the group of individuals without shrinkage, thus impairing the physiological function of SMCs and increasing inflammation. It is well known that both hypertension and smoking significantly affect endothelial cells (ECs) and SMCs [21,22]. In contrast, in patients with shrinkage, the regeneration of NRGN seemed to be more efficient, reflecting the fate of vascular cells and the extent of aortic wall remodelling. Consequently, NRGN might serve as a new potential biomarker of aortic sack shrinkage/expansion.

Casein alpha s1 (CSN1S1)

Adjustment for statins led to a significant reduction in NRGN as well as an increase in CSN1S1. These results seem to be contradictory because statins have already been well described as an effective drug against inflammation in AAAs [23]. However, statins also affect SMC proliferation and increase apoptosis, circumstances that might weaken the aortic wall [24]. Caseins are the main proteins of human milk and can have various biological effects, including the modulation of inflammation, chemotaxis, and cell growth [25,26,27]. Recently, CSN1S1 expression was found in the lymph nodes and blood of multiple sclerosis patients [28]. Furthermore, other studies reported that CSN1S1 influences the differentiation of monocytes into macrophages and upregulates the expression of various pro-inflammatory cytokines, such as IL-1β [27,29]. Thus, an increase in CSN1S1, along with the reduced levels of NRGN, might reflect the inflammatory state in the aortic wall of the EVAR patients and the extent of aortic wall remodelling.

Interestingly, the statistical adjustment for the preoperative aortic sac diameter showed the most significant differences in the abundance of proteins in plasma between the study patients. Consequently, the extent of aortic dilation seems to be the critical factor reflected in the affected patient’s blood.

Calpastatin

The differentially occurring calpastatin, for instance, specifically inhibits the proteolytic activity of calpain (calcium-dependent cysteine protease). Calpains are ubiquitous non-lysosomal cysteine proteases, which are activated by calcium signalling. Recently, it has been demonstrated that the expression of calpain was increased in aneurysm tissues obtained from Marfan syndrome patients, whereas calpastatin was decreased [30]. The changes in calpain expression modulate the structure of aortic tissue, causing an alteration in elastin structure, thus enabling macrophage infiltration and elevation of matrix metalloproteinases (MMP) levels. Furthermore, circulating levels of calpain and, thus, calpastatin might be used for the prognosis of aortic wall remodelling in patients post-EVAR. Further evidence suggests that a dysfunctional calpain proteolytic system, e.g., through increased inhibition by calpastatin, as we observed in our study, contributes to erythrocyte (EC) disorder, leading, among other things, to the disorganisation of cell–cell junctions and dysfunction of nitric oxide synthase.

SCUBE (signal peptide, complement C1r/C1s, Uegf, Bmp1 (CUB) domain, and epithelial growth factor (EGF)-like domain-containing protein)

The SCUBE (signal peptide, complement C1r/C1s, Uegf, Bmp1 (CUB) domain, and epithelial growth factor (EGF)-like domain-containing protein) family consists of three highly conserved proteins, SCUBE1, 2, and 3. The SCUBE expression has been found in ECs, platelets, epithelium, and osteoblasts. The human SCUBE functional analysis from genetically modified mouse models has already been described in the context of cancer, skeletal disease, and cardiovascular diseases.

A soluble SCUBE1 is released, for instance, from activated platelets and has been described as a clinical biomarker for acute coronary syndrome and ischemic stroke [31]. Ali et al. have reported, in addition, that SCUBE2 and 3 might also play a role in cardiovascular disorders and atherosclerosis [32]. In an atherosclerotic LDLr-/- mouse model, the SCUBE2 expression markedly increased after the start of the high-fat diet. These findings were also confirmed in human coronary arteries with advanced atherosclerotic plaques. Yang et al. demonstrated that SCUBE3 may also play a vital role in regulating cardiac and vascular remodelling responses through stabilising the TGF-beta1 signalling pathway [33]. In our experimental setting, SCUBE3 significantly increased in the non-shrinkage patient group, with the suspicion of increased atherosclerotic changes in the aortic wall in these patients compared to patients with shrinkage.

The ubiquitin–proteasome system is involved in numerous cellular processes, cell growth, and homeostasis, removing abnormal and short-lived peptides from the cellular circulation. The system consists of ubiquitin (Ub), ubiquitin-activating enzyme (E1), ubiquitin-conjugating enzyme (E2), ubiquitin-ligase enzyme (E3), 26S proteasome, deubiquitinating enzymes (DUBs), and target proteins [34]. Ubiquitin-conjugating enzymes are responsible for the second step in the ubiquitination of target protein degradation via the proteasome [35]. Lately, ubiquitin-conjugating enzyme E2 (UBE2) has been reported to be differentially expressed in individuals with abnormal coronary endothelial function [36]. Furthermore, UBE2 was also observed to inhibit the proliferation and migration of human vascular smooth muscle cells. In our study, a significantly lower (>20-fold) abundance of UBE2 was observed in EVAR patients with aortic sac shrinkage. How far these changes could be relevant has to be a subject of further investigations.

Further proteins

The potential role of the other significantly differentially abundant proteins, keratinocyte proline-rich protein (KPRP), loricrin, prolactin-inducible protein (PIP), serpin B12, and skin-specific protein 32 (C1orf68) in plasma of patients after EVAR treatment following adjustment for aortic diameter is less clear and has to be confirmed in a larger study. Proline-rich proteins (PRPs) contain several repeats of short proline-rich sequences. They have been described particularly in salivary excretion [37]. There is no knowledge about the role, especially keratinocyte PRPs (KPRPs) in cardiovascular diseases. Loricrin is the main structural protein of the cornified envelope. In humans, loricrin is expressed in mammalian epithelia with the highest levels of expression in the epidermis, oral and anal mucosa, oesophagus, foreskin, and vagina [38]. The reason we observed differences in the level of loricrin in our study patients has to be clarified in further studies. The PIP is a small protein regarded as a marker of mammary differentiation. It is highly expressed in the mammary gland and breast cancer. Current studies suggest its primary role in host defence and immune response [39]. Furthermore, PIPs have been described as structural proteins in epithelial cells and keratinocytes [40]. Loricrin is a major protein component of the cornified cell envelope and is mainly found in epidermal cells [41]. Skin-specific protein 32 (C1orf68) has been found particularly in the cytoplasm of skin cells and shows the highest enrichment in the skin [42]. The serine protease inhibitor (serpin) protein superfamily is involved in various biological functions, including matrix remodelling, fibrinolysis, thrombosis, and tumour progression [43]. Serpin B12 is an inhibitor of trypsin and plasmin. The expression of serpin B12 in epithelia and macrophages suggests a potential role in host defence via the inhibition of various proteases. Whether serpin B12 can also play a role in aortic sack expansion/shrinkage is so far unknown. C1orf68 is a human gene encoding the skin-specific protein 32. C1orf68 is expressed in the skin as a part of the epidermal differentiation complex.

Interestingly, the above-described proteins with significant differences between the study groups, KPRP, loricrin, PIP, SB12, and C1orf68 are all more or less associated with epithelia and epidermis. How far it is just a coincidence related to the selection of our small study groups, or whether any causal relationships can be drawn, remains to be elucidated in further studies.

Limitations

However, some shortcomings of this study must also be mentioned. Firstly, this is a very small study population, with only 32 patients over 11 years who met the inclusion and exclusion criteria and the different sizes of the study groups, with 20 patients for the shrinkage cohort and only 12 patients for the stable/expansion cohort. In addition, only men were eligible for inclusion in this study. The time at which the sac behaviour was evaluated, and the two groups were defined accordingly, was 2 years after surgery. However, due to the study’s retrospective nature, the time of blood sampling for the proteomics analysis was significantly later, with a median value of 44 months postoperatively.

## 5. Conclusions

This study utilised proteomic analysis to identify biomarkers associated with sac shrinkage or expansion in patients undergoing endovascular aneurysm repair for abdominal aortic aneurysms. The findings revealed that distinct protein profiles, including neurogranin, casein alpha S1, and ubiquitin-conjugating enzyme E2, may be correlated to aneurysm sac behaviour and, therefore, potentially influence EVAR outcomes. Further research is necessary to understand the underlying biological mechanisms and to validate these biomarkers in larger cohorts, including studies with patients who have endoleaks.

## Figures and Tables

**Table 1:** Patient characteristics. (n.s.: non-significant; COPD: chronic obstructive pulmonary disease; CKD: chronic kidney disease; CVD: cardiovascular disease; OP: operation; FU: follow-up).

	Overall	Shrinkage (*n* = 20)	Stable/Expansion (*n* = 12)	*p*-Value *
Age (years, median (range))	73 (56–89)	74 (56–89)	71 (57–88)	n.s.
Sex (male)	100%	20 (100%)	12 (100%)	n.s.
Time To Blood Sampling (median (range))	44 (13–104)	50 (14–104)	23 (13–82)	0.020
Comorbidities				
Hypertension	30 (94%)	18 (90%)	12 (100%)	n.s.
Hyperlipidemia	26 (81%)	17 (85%)	9 (75%)	n.s.
Smoking	29 (91%)	19 (95%)	10 (83%)	n.s.
COPD	8 (25%)	6 (30%)	2 (17%)	n.s.
CKD	8 (25%)	5 (25%)	3 (25%)	n.s.
CVD	15 (47%)	9 (45%)	6 (50%)	n.s.
Medication				
Antiplatelet Therapy	27 (84%)	18 (90%)	9 (75%)	n.s.
Anticoagulation	10 (31%)	5 (25%)	5 (42%)	n.s.
Beta-Blocker	14 (44%)	9 (45%)	5 (42%)	n.s.
Statins	30 (94%)	19 (95%)	11 (92%)	n.s.
Operative Details				
Maximum Diameter OP (mm, median (range))	59 (44–91)	60 (47–91)	58 (44–73)	n.s.
Maximum Diameter at last FU (mm, median (range))	48 (35–85)	47 (37–85)	57 (35–67)	0.026
Stent Grafts				n.s.
Medtronic Endurant II	10 (31%)	8 (40%)	2 (17%)	
Cook Zenith Alpha	7 (22%)	4 (20%)	3 (25%)	
Gore Excluder	10 (31%)	7 (35%)	3 (25%)	
Lombard Minos	5 (16%)	1 (5%)	4 (33%)	

* For continuous variables, non-parametric Mann–Whitney U-test was used; for nominal variables, Fisher’s exact test was applied. Abbreviation: n.s., non significant

**Table 2:** Selected differentially abundant proteins in plasma samples in EVAR patients with and without shrinkage. (NRGN: neurogranin; CSN1S1: casein alpha s1; SCUBE3: signal peptide, complement C1r/C1s, Uegf, Bmp1 (CUB) domain, and epithelial growth factor (EGF)-like domain-containing protein; KPRP: keratinocyte proline-rich protein; PIP: prolactin-inducible protein, (F) serpin B12; C1orf68: skin-specific protein 32; UBE2: ubiquitin-conjugating enzyme E2 (UBE2); FDR: false discovery rate. * Significant differences—FDR < 0.1).

	Protein_ID	Q92686	P02662	P20810	Q8IX30	Q5T749	P23490	P12273	Q96P63	Q5T750	Q13404
	Protein Name	NRGN	CSN1S1	Calpastatin	SCUBE3	KPRP	Loricrin	PIP	Serpin B12	C1orf68	UBE2
Without adjustment	FDR	0.190	0.701	0.443	0.443	0.443	0.443	0.534	0.551	0.551	0.826
	*p*-value	0.000	0.062	0.005	0.005	0.006	0.007	0.012	0.015	0.017	0.232
	fold change	−17.42	0.613	1.86	−1.33	1.82	1.95	1.60	1.84	2.08	−1.98
Diameter	FDR	0.164	0.203	0.050 *	0.075 *	0.050 *	0.050	0.050 *	0.089 *	0.050 *	0.050 *
	*p*-value	0.004	0.006	<0.001	0.001	<0.001	<0.001	<0.001	0.001	<0.001	<0.001
	fold change	−11.37	0.846	2.65	−1.47	2.22	2.62	1.89	2.37	3.31	−22.23
Hypertension	FDR	0.025 *	0.915	0.859	0.859	0.780	0.780	0.780	0.780	0.719	0.895
	*p*-value	<0.001	0.182	0.099	0.060	0.010	0.021	0.006	0.013	0.014	0.201
	fold change	−13.50	0.610	1.57	−1.28	1.97	2.04	1.81	2.15	2.05	−2.07
CAVK	FDR	0.023 *	0.793	0.502	0.518	0.502	0.502	0.603	0.603	0.518	0.929
	*p*-value	<0.001	0.052	0.006	0.008	0.004	0.006	0.017	0.016	0.008	0.408
	fold change	−13.50	0.644	1.83	−1.32	4.72	1.96	1.51	1.79	−1.32	−1.60
Smoking	FDR	0.025 *	0.952	0.755	0.888	0.952	0.755	0.952	0.952	0.796	0.952
	*p*-value	<0.001	0.432	0.005	0.014	0.045	0.005	0.059	0.039	0.012	0.263
	fold change	−13.50	0.322	2.12	0.74	1.60	2.25	1.54	1.79	2.39	−1.94
Statins	FDR	0.025 *	0.049 *	0.635	0.698	1.000	0.856	0.542	1.000	1.000	0.863
	*p*-value	<0.001	<0.001	0.016	0.025	0.103	0.045	0.010	0.261	0.163	0.195
	fold change	−13.50	1.862	1.90	−1.37	1.55	1.92	1.80	1.42	1.69	−2.07

## Data Availability

The data presented in this study are available upon request from the corresponding author; the data are not publicly available due to ethical restrictions.
